# Pooled Analysis of Central Venous Pressure and Brain Natriuretic Peptide Levels in Patients With Extubation Failure

**DOI:** 10.3389/fphys.2022.858046

**Published:** 2022-07-15

**Authors:** Jianghong Cao, Beibei Wang, Lili Zhu, Lu Song

**Affiliations:** ^1^ Department of Intensive Care Unit, Shanxi Provincial People’s Hospital, Taiyuan, China; ^2^ Department of Cardiology, The First People’s Hospital of Jinzhong, Jinzhong, China

**Keywords:** central venous pressure, brain natriuretic peptide, extubation failure, pooled-analysis, cardiac insufficiency

## Abstract

**Purpose:** Cardiac insufficiency has been considered to be a common cause of extubation failure. Some studies have shown that central venous pressure (CVP) and brain natriuretic peptide (BNP) are able to predict extubation outcomes. Therefore, we conducted a pooled analysis to evaluate the potential of CVP and BNP levels as predictors of extubation outcomes, using a cohort of critically ill patients who were on mechanical ventilation (MV).

**Methods:** We searched three online electronic databases up to October 2021. All data were analyzed using Review Manager 5.4. For each study, the analysis was performed using standardized mean differences (SMD) with 95% confidence intervals (CI).

**Results:** The pooled analysis of seven studies on CVP levels and extubation outcomes showed that elevated CVP levels were significantly associated with extubation failure (SMD:0.47, 95% CI: 0. 43–0.51, *p* < 0.00001). This association also appeared before extubation (SMD:0.47, 95% CI: 0. 43–0.51, *p* < 0.00001), but it did not appear after extubation (SMD: 0.63, 95% CI: −0.05–1.31, *p*=0.07). Similarly, pooled analysis of eight studies on BNP levels and extubation outcomes showed that increased BNP levels are closely related to extubation failure (SMD:0.68, 95% CI: 0.49–0.86, *p* < 0.00001). This relationship also occurs before (SMD: 0.57, 95% CI: 0.35–0.79, *p* < 0.00001) and after (SMD: 0.91, 95% CI: 0.59–1.23, *p* < 0.00001) extubation.

**Conclusions:** This study showed that elevated CVP and BNP levels are associated with extubation failure in critically ill patients. However, BNP levels are more valuable than CVP levels in predicting extubation outcomes.

## Introduction

Liberation from mechanical ventilation (MV) is a very challenging process for clinicians in the Intensive Care Unit (ICU). Premature extubation is associated with the probability of reintubation, extended ICU stay, and mortality (Harrison et al., 2002; Perren et al., 2010). Delayed extubation may lead to ventilator-acquired pneumonia, prolonged hospital stay, and high mortality (Esteban et al., 2002; Thille et al., 2011; Fernandez-Zamora et al., 2018). Therefore, it is extremely important to identify reliable and accurate markers for predicting extubation outcomes.

Traditionally, respiratory failure was considered to be the main cause of extubation failure. Consequently, predictive markers for extubation outcomes have focused primarily on breathing-related parameters, including rapid shallow breathing index (f/Vt), respiratory rate, minute ventilation, and cough intensity (Khamiees et al., 2001; Frutos-Vivar et al., 2006). Unfortunately, these predictors do not accurately predict extubation outcomes (Hsieh et al., 2018). Recently, other studies have shown that cardiac dysfunction plays an important role in extubation outcomes, and respiratory diseases have a certain impact on cardiac function (Chatila et al., 1996; Frazier et al., 2006). Moreover, some reports have shown that cardiac insufficiency is a cause of failure in as many as 50% of patients who have failed extubation. Thus, a simple and effective method is urgently needed to predict extubation outcomes for those with cardiac dysfunction.

Central venous pressure (CVP) reflects right atrial force, which is influenced by cardiac function, blood volume and vascular tension. Brain natriuretic peptide (BNP) is mainly secreted by cardiomyocytes to compensate for myocardial stretch and volume overload. Both CVP and BNP are important markers for monitoring cardiac function and volume, which can be affected by disconnection from MV. Previous studies have shown that the predictive value of BNP levels on extubation outcomes is controversial. There are few studies on the predictive value of CVP levels on extubation outcomes. Thus, we systematically reviewed the literature and conducted a pooled analysis to determine the association and predictive value of BNP and CVP levels on extubation outcomes.

## Materials and Methods

### Search Strategy

To identify qualified published studies, two independent researchers (ZLL and SL) systematically searched the Web of Science, EMBASE, and Cochrane Library, using the following keywords: “extubation,” “weaning,” “disconnect of mechanical ventilation,” “discontinuation of mechanical ventilation,” “central venous pressure,” “CVP,” “Brain natriuretic peptide,” “BNP,” and “brain natriuretic peptide”. The searches encompassed studies up to October 2021 and were limited to those published in English. In addition, we manually searched relevant reviews and references within those publications to identify potentially relevant studies. If there was a disagreement about inclusion, a third researcher (CJH) was called to discuss the decision.

### Inclusion and Exclusion Criteria

The inclusion criteria were as follows: patients were adults hospitalized in the ICU for underlying cardiovascular or respiratory disease who received MV for no less than 24 h. Patients were extubated after adequate evaluation and followed for at least 24 h after weaning from MV. CVP or BNP levels were monitored before or after extubation. Extubation failure was defined as re-intubation within 48 h, spontaneous respiratory failure, noninvasive or invasive ventilation within 48 h after extubation, or death within 48 h. The exclusion criteria were as follows: potential confounding variables for CVP, such as lax measurement of CVP, potential confounding factors for BNP such as renal insufficiency, studies lacking the necessary data, duplicate studies, reviews, case reports, abstracts or letters.

### Data Extraction and Quality Assessment

Two researchers (ZLL and SL) independently screened the titles and abstracts using set keywords, and then checked the full text according to inclusion and exclusion criteria. The following information was extracted: the first author’s name, publication year, age, sex (%), country, sample size, timing of BNP and CVP measurement, methods and durations of SBT and definition of extubation failure. When there were differences in data extraction, it was discussed with the third researcher (CJH). The Newcastle-Ottawa scale (NOS) was used to assess the quality of the included studies (Stang, 2010). Studies that had a score ≥6 points were considered a “high-quality study.” This study was approved by the Ethics Committee of Shanxi Provincial People's Hospital (No. 223, 2022).

### Statistical Analysis

The pooled analysis was analyzed by Review Manager 5.4. Standardized mean differences (SMD) and corresponding 95% confidence intervals (CIs) of the CVP and BNP levels were collected and calculated for each study. When the median and interquartile range (IQR) was provided, the mean and standard deviation (M ± SD) was estimated using Luo’s approach and Wan’s method, respectively (Wan et al., 2014; Luo et al., 2018). Heterogeneity is evaluated by calculating the *I*- squared (*I* ^2^) index. *I* ^2^ values of 75%–100%, 50%–75%, 25%–50% and < 25% were considered as high, moderate, low heterogeneous and homogeneous, respectively. If there was significant heterogeneity (*I* ^2^ > 50% or *p* < 0.05), a random effect model was used, otherwise, a fixed effect model was applied.

In order to identify the potential heterogeneity, we performed subgroup analysis based on the levels of CVP and BNP before and after extubation. Moreover, we deleted one study at a time and repeated the analysis, namely the leave-one-out method for sensitivity analysis. Egger’s and Begg’s tests were used to find potential publication bias. When the *p* value was less than 0.05, it was considered statistically significant.

## Results

### Study Processing

**FIGURE 1:**
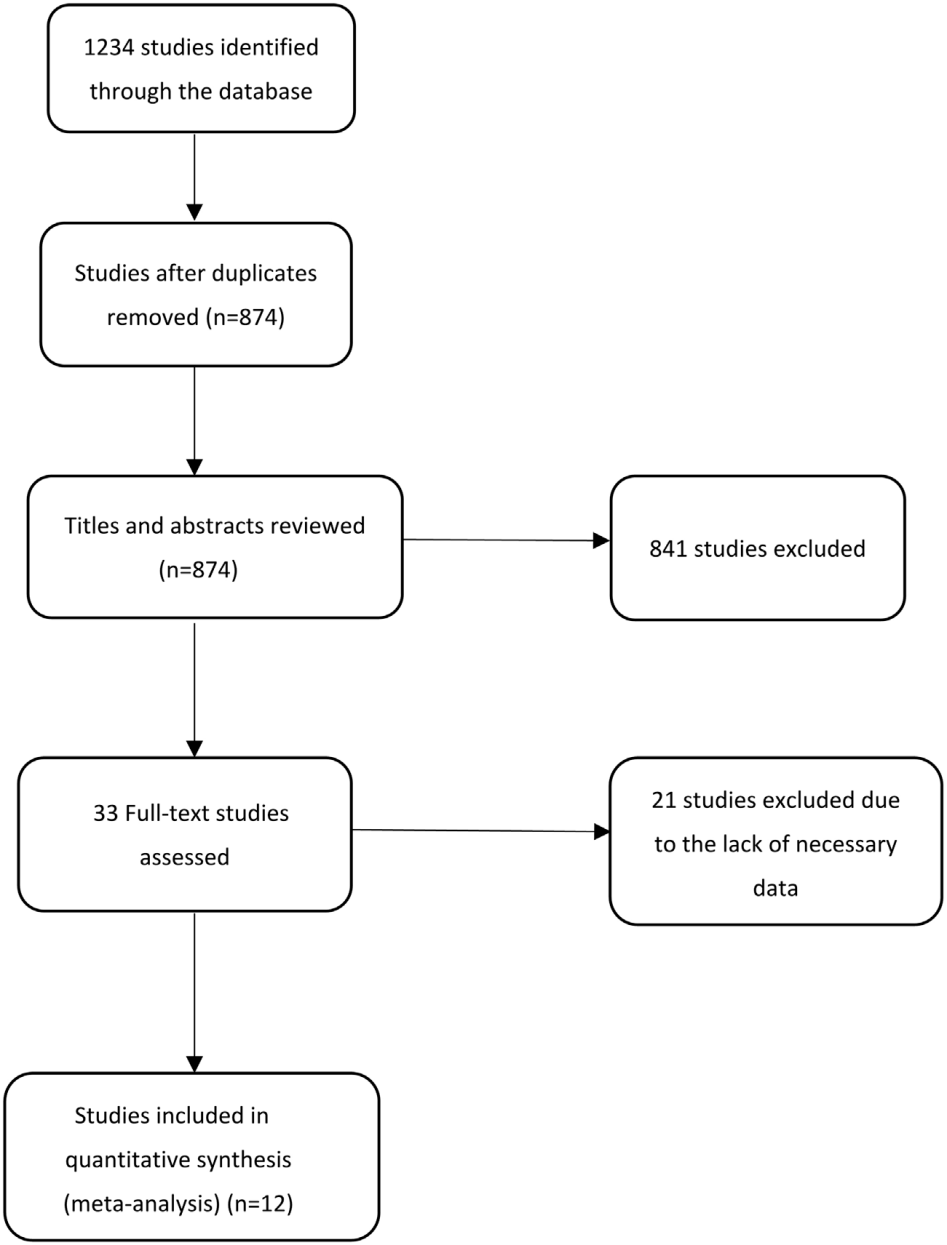
Selection process for studies included in the pooled- analysis.

Using our search strategy, a total of 1,234 potentially original studies were identified and excluding duplications, 874 studies remained. Careful screening of titles and abstracts, identified 841 studies that did not meet the inclusion and exclusion criteria, and these were also excluded. Finally, after carefully reading the main body of the remaining 33 studies, 20 studies were eventually excluded. In the end, 12 qualified studies were inclued (Chien et al., 2008; Zapata et al., 2011; Saugel et al., 2012; Soummer et al., 2012; Ma et al., 2013; Maraghi et al., 2014; Farghaly et al., 2015; Konomi et al., 2016; Tanios et al., 2016; Haji et al., 2018; Dubo et al., 2019; Zhao et al., 2021). The screening process of these studies is shown in Figure 1. Meanwhile, Table 1 listed the basic characteristics of the included studies. As NOS scores of these studies were at least 6, all were considered of high quality.

**TABLE 1:** Characteristics of studies.

Study	Country	Age (ES/EF)	Male (%)	Setting	N (ES/EF)	Indicator	Timing of extubation	Methods and durations of SBT	Definition of EF	NOS
Chien2008 testing group (Chien et al., 2008)	China	79.1 ± 8.6/81.5 ± 7.4	53	ICU	33/8	BNP	Before extubation	2 h SBT	SBT failure or reintubation within 48 h	7
Chien2008 validation group (Chien et al., 2008)	China	79.7 ± 9.2/76.3 ± 7.3	57	ICU	38/11	BNP	Before extubation	2 h SBT	SBT failure or reintubation within 48 h	7
Dubo 2019 (Dubo et al., 2019)	Chile	54 ± 21/47 ± 24	81	medical-surgical ICU	154/11	CVP	Before and after extubation	T- piece and 60–120 min SBT	Reintubation within 48 h	7
Konomi 2016 (Konomi et al., 2016)	Greece	54 ± 21/69 ± 16	40	Multidisciplinary ICU	27/15	CVP and BNP	Before and after extubation	T- piece and 2 h SBT	SBT failure or reintubation within 48 h	6
Ma 2013 (Ma et al., 2013)	China	62.3 ± 12.0/59.1 ± 3.9	85.7	ICU	22/7	CVP	Before extubation	T- piece and 120 min SBT	Reintubation within 48 h	6
Saugel 2012 (Saugel et al., 2012)	Germany	63.5 ± 14.5/64.6 ± 8.4	86	medical ICU	54/7	CVP	Before extubation	NG	Reintubation within 48 h	7
Zapata 2011 (Zapata et al., 2011)	Spain	61.6 ± 14.7/66.7 ± 9.7	68	ICU	58/10	CVP and BNP	Before and after extubation	T- piece and 30–120 min SBT	Reintubation within 48 h	8
Zhao 2021 MIMIC-IV (Zhao et al., 2021)	NG	64 ± 16/68 ± 15	NG	ICU	13,433/2,756	CVP	Before extubation	NG	Reintubation	7
Zhao 2021 ZS (Zhao et al., 2021)	China	60 ± 13/63 ± 12	NG	ICU	451/51	CVP	Before extubation	NG	NIV、 reintubation or death within 48 h	8
Farghaly 2015 (Farghaly et al., 2015)	Australia	53.81 ± 18.9/57.14 ± 12.9	43	Respiratory ICU	16/14	BNP	Before and after extubation	PSV and 2 h SBT	SBT failure or reintubation within 48 h	8
Maraghi 2014 (Maraghi et al., 2014)	Egypt	46 ± 10.35/54 ± 9.25	32	ICU	25/7	BNP	Before and after extubation	T-piece and 2 h SBT	SBT failure or reintubation within 48 h	6
Soummer 2012 (Soummer et al., 2012)	France	59 ± 14± 15	59	Multidisciplinary ICU	57/29	BNP	Before and after extubation	T- piece and 60- min SBT	Noninvasive or invasive ventilation) within 48 h after extubation	7
Tanios 2016 (Tanios et al., 2016)	United States	NA	48	ICU	56/29	BNP	Before extubation	PSV and 2 h SBT	SBT failure	6
Haji 2018 (Haji et al., 2018)	Australia	63.5 ± 4.6/77 ± 2.7	64	ICU	42/11	BNP	after extubation	PSV and 60min SBT	Nonscheduled NIVM, or death within 48 h	7

Abbreviations: EF, extubation failure; ES, extubation successI; ICU, intensive care unit; SBT, spontaneous breathing trail; BNP, brain natriuretic peptide; CVP: central venous pressure; NG: not given.

### Central Venous Pressure Levels and Extubation Outcomes

**FIGURE 2:**
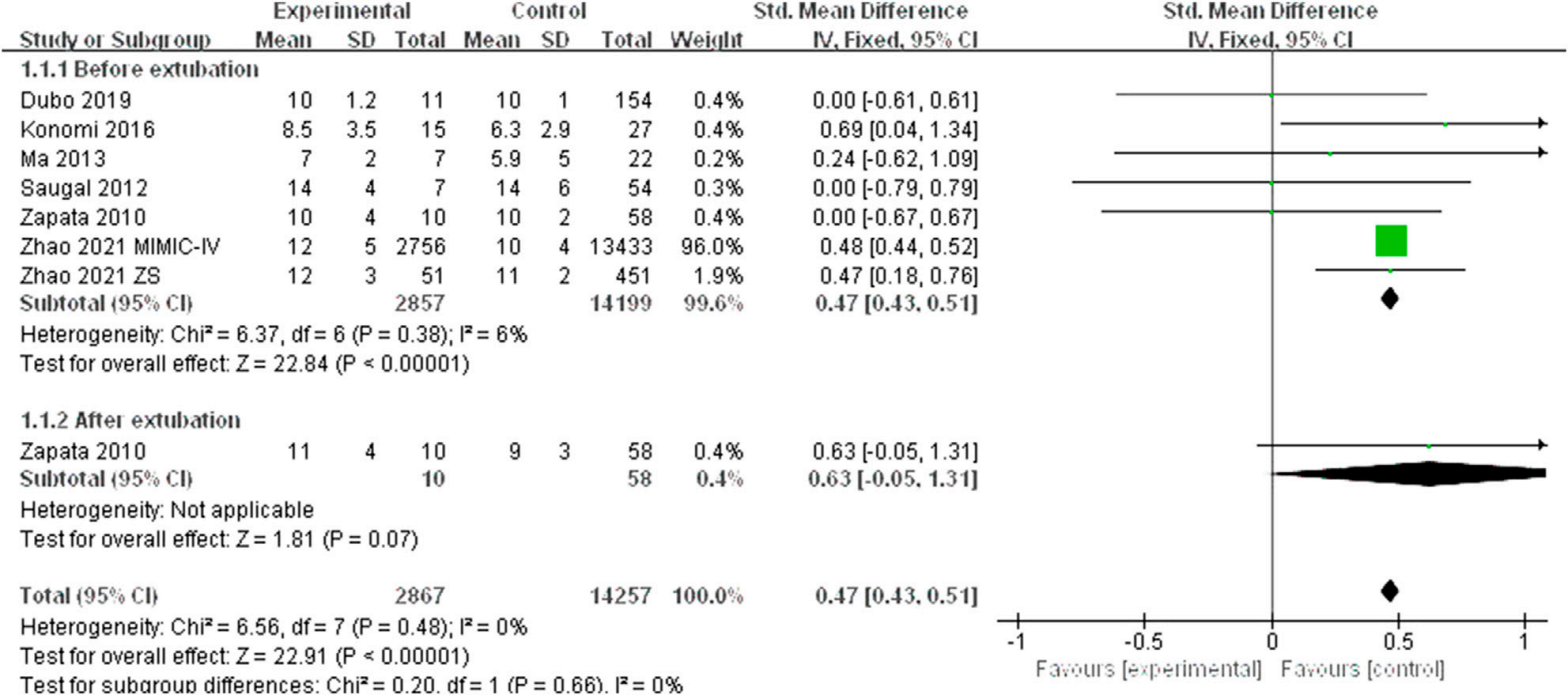
The CVP levels standard mean difference between extubation failure and extubation success groups.

We found seven studies that described a relationship between the CVP levels and extubation outcomes. Since there was no significant heterogeneity (*I* ^2^ < 50%), the fixed effect model was applied. The fixed effect pooled SMD was 0.47 (95% CI: 0.43–0.51, *p* < 0.00001) (Figure 2). Subgroup analysis based on extubation time showed that the results of pooled analysis were consistent with the association between elevated CVP levels and extubation failure before extubation (SMD:0.47,95% CI: 0.43–0.51, *p* < 0.00001). However, this association had no significant correlation after extubation (SMD: 0.63, 95% CI: −0.05–1.31, *p*=0.07).

Due to the limited studies included, potential publication bias was not carried out. Since none of the studies had a significant influence on the pooled results, the leave-one-out sensitivity analyses suggested robust results.

### Brain Natriuretic Peptide Levels and Extubation Outcomes

**FIGURE 3:**
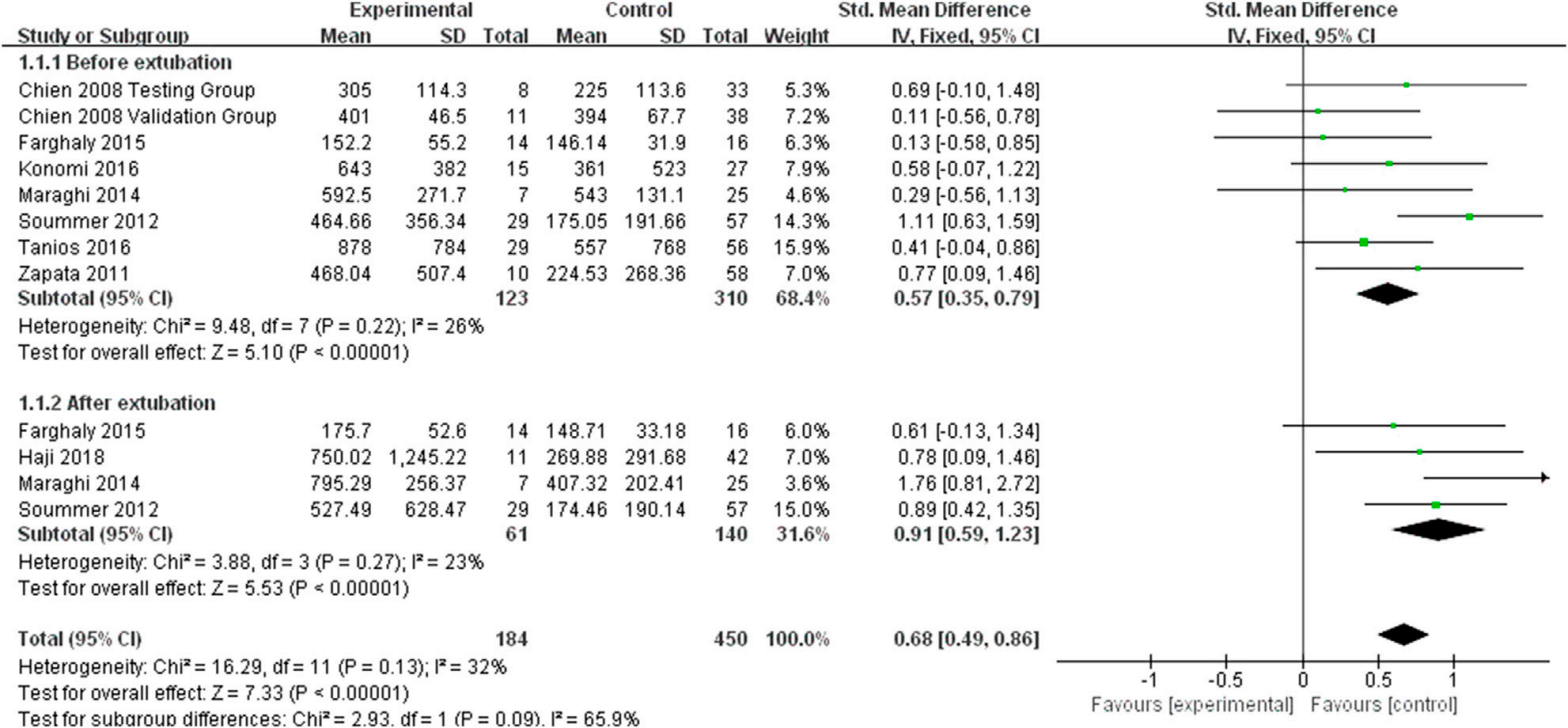
The BNP levels standard mean difference between extubation failure and extubation success groups.

We also found eight studies that contained information about the association between the BNP levels and extubation outcomes. Since there was no significant heterogeneity (*I* ^2^ < 50%), the fixed effect model was applied. The fixed effect pooled SMD was 0.68 (95% CI: 0.49–0.86, *p* < 0.00001) (Figure 3). Based on the subgroup analysis of extubation time, we found that the pooled results were consistent with the relationship between high BNP levels and extubation failure before extubation (SMD: 0.57, 95% CI: 0.35–0.79, *p* < 0.00001) and after extubation (SMD: 0.91, 95% CI: 0.59–1.23, *p* < 0.00001).

No potential publication bias was observed with respect to the relationship between BNP levels and extubation outcomes, according to the results of the Egger’s test (*P* > 0.05) and Begg’s test (*P* > 0.05). The leave-one-out sensitivity also showed robust results, as none of the studies had a significant effect on the overall outcomes.

## Discussion

In this study, we identified an association between high CVP and BNP levels with extubation failure in critically ill patients. An association was also found with both elevated BNP and CVP levels before extubation and elevated BNP levels after extubation. However, this association did not appear in elevated CVP levels after extubation. Thus, these results suggested that the measurement of CVP and BNP levels may be useful indicators for patients undergoing extubation from MV. Moreover, compared with CVP levels, BNP levels are a more valuable predictor of extubation outcomes. The values of *I* ^2^ = 0% for CVP and *I* ^2^ = 23% for BNP showed that our pooled analysis was homogeneous, and the leave-one-out sensitivity showed our results are robust.

In the process of liberation from MV, the intrathoracic pressure develops from positive to negative. This promotes the systemic venous blood reflux increases the right ventricular preload and augmented left ventricular transmural pressure and afterload (Buda et al., 1979). Moreover, emotional stress and potential hypoxia during extubation may lead to sympathetic excitation. Many critically ill patients have undiagnosed or subclinical cardiovascular diseases, which often hiderfluid management and cardiovascular compensation. Thus, insufficient cardiac reserve may lead to subsequent respiratory insufficiency and failed extubation (Jubran et al., 1998). In particular, marked or even asymptomatic, diastolic or systolic dysfunction, as well as arrhythmias, coronary heart disease, and other heart conditions increase the likelihood of extubation failure.

Both CVP and BNP levels are able to monitor heart function and volume. Critically ill patients in the ICU often require an indwelling central venous catheter to monitor cardiac function and fluid replacement. There are only a few studies describing the association between CVP levels and extubation outcomes. Our analysis suggested that elevated CVP levels are associated with extubation failure, and that this association was observed before extubation. This result is consistent with the results of Dubo et al. (2019), which demonstrated that an early rise in CVP levels before extubation, increased the risk of extubation failure. However, this association was not observed after extubation. We believe that this conclusion is controversial for two main reasons: first, after adequate treatment, most patients’ cardiac function improved significantly after extubation. Potential cardiac insufficiency could not be detected due to the poor sensitivity of CVP in monitoring cardiac function and volume. Second, the lack of data may be one reason for conflicting results, as only one studies were included. Therefore, more well-designed studies should be carried out.

BNP has been proven to be a sensitive serum marker of cardiac dysfunction (Win et al., 2005). Zapata et al. (2011) and Lara et al. (2013) found compared with patients with successful extubation, patients with extubation failure had higher BNP levels before and after extubation. Our study also confirmed that elevated BNP levels were significantly associated with extubation failure. Our anlaysis also showed that elevated BNP levels before extubation were closely related to extubation failure, consistent with studies by Chien et al. and Konomi et al. Unlike CVP, this association also appeared after extubation. Some studies have shown that increased BNP levels after extubation are a predictive factor for extubation failure (Lemaire et al., 1988; Jubran et al., 1998). Since BNP is a sensitive indicator of cardiac insufficiency, potential cardiac insufficiency can be detected early on.

### Strengths and Limitations

The measurement of CVP levels is affected by the observer’s mode of measurement and the patient’s condition. For example, intra-thoracic pressure, intra-abdominal pressure, position, or depth of venous catheter placement can affect the results. Although we can standardize the observer’s measurement and try to avoid a patient influence on the CVP measurements, there is poor predictive value for extubation outcomes. As ICU patients often have in-dwelling central venous catheters, CVP could easily and quickly be evaluated at the bedside. BNP is a sensitive marker for monitoring cardiac insufficiency and accurately predicts extubation outcomes. However, the utility of BNP as a marker also has its limitations. The detection of BNP is affected by age, renal insufficiency, drugs and other unavoidable factors. In conclusion, although CVP is able to identify early extubation outcomes, its predictive value is poor. In contrast, BNP has high predictive value, but the detection of extubation outcomes is inferior. Finally, the accuracy of our results may be affected by the limited number of studies we included. Since we do not have access to the original data for drawing ROC curves, we cannot determine a reliable cut-off point fot the CVP and BNP tests.

## Conclusion

Our study showed that elevated CVP and BNP levels are related to the risk of extubation failure. More importantly, compared with CVP levels, BNP levels are more valuable than CVP levels in predicting extubation outcomes.

## Data Availability

The original contributions presented in the study are included in the article/Supplementary Material, further inquiries can be directed to the corresponding author.
